# HGTree: database of horizontally transferred genes determined by tree reconciliation

**DOI:** 10.1093/nar/gkv1245

**Published:** 2015-11-17

**Authors:** Hyeonsoo Jeong, Samsun Sung, Taehyung Kwon, Minseok Seo, Kelsey Caetano-Anollés, Sang Ho Choi, Seoae Cho, Arshan Nasir, Heebal Kim

**Affiliations:** 1 Interdisciplinary Program in Bioinformatics, Seoul National University, Kwan-ak St. 599, Kwan-ak Gu, Seoul, 151-741, Republic of Korea; 2 Department of Animal Sciences, University of Illinois, Urbana, IL 61801, USA; 3 C&K genomics, Main Bldg. #514, SNU Research Park, Seoul 151-919, Republic of Korea; 4 Department of Agricultural Biotechnology, Seoul National University, Seoul 151-742, Republic of Korea; 5 National Research Laboratory of Molecular Microbiology and Toxicology, Department of Agricultural Biotechnology, Center for Food Safety and Toxicology, Seoul National University, Seoul 151-921, Republic of Korea; 6 Department of Biosciences, COMSATS Institute of Information Technology, Park Road, Chak Shahzad, Islamabad 45550, Pakistan

## Abstract

The HGTree database provides putative genome-wide horizontal gene transfer (HGT) information for 2472 completely sequenced prokaryotic genomes. This task is accomplished by reconstructing approximate maximum likelihood phylogenetic trees for each orthologous gene and corresponding 16S rRNA reference species sets and then reconciling the two trees under parsimony framework. The tree reconciliation method is generally considered to be a reliable way to detect HGT events but its practical use has remained limited because the method is computationally intensive and conceptually challenging. In this regard, HGTree (http://hgtree.snu.ac.kr) represents a useful addition to the biological community and enables quick and easy retrieval of information for HGT-acquired genes to better understand microbial taxonomy and evolution. The database is freely available and can be easily scaled and updated to keep pace with the rapid rise in genomic information.

## INTRODUCTION

Vertical inheritance refers to the transfer of genetic information from parents to offspring. Vertically inherited genes typically show higher degree of similarity between species that are closely related than those that are distantly related. This aids in reliable recognition of species and understanding their classification and evolution. For example, ribosomal RNA (rRNA) genes have been historically used to determine the taxonomic structure of cellular life (1). However, vertical signal can sometimes be confounded by acquisition of genes from other sources such as environment, viruses, or via direct interactions between organisms. Recent advances in genomics have confirmed the existence of ‘foreign’ genes embedded in cellular genomes. For example, mammalian genomes are enriched with viral-like genetic elements, constituting up to 8% of the human genome (2). Similarly, many microbial genomes possess genes acquired from multiple sources (3). This phenomenon is referred to as horizontal gene transfer (HGT), which is a natural outcome given the numerous ways species interact with each other and occupy common habitats.

HGT allows gain of novel molecular functions and can provide selective evolutionary advantages to species. For example, transfer of antibiotic resistance and virulence factor genes between bacterial species poses significant challenges to human health (4). Similarly, transfer of genes involved in response to heat and cold shock and heavy metal and ultra-violet resistance facilitates bacterial adaptation to certain environments. While HGT is an important force driving the evolution of (especially) microbial organisms (5), it can complicate interpreting the true evolutionary history of species and can lead to erroneous interpretations regarding their classification and community interactions (3). Therefore, it is crucial to distinguish between vertically and horizontally acquired genes in genomes, especially when studying deep evolutionary relationships.

Accurate detection of HGT however remains a computational and conceptual challenge. Existing databases such as HGT-DB (6) and DarkHorse HGT Candidate Resource (7) use genomic signatures (i.e. GC bias, nucleotide composition and codon usage) and implicit phylogenetic methods (i.e. comparing the evolutionary distance inferred from sequence similarity) to detect HGT. Because genomic signatures of transferred genes may lose their ‘distinctiveness’ over long periods of evolutionary time and tend to be highly similar to host genomes in cases of HGT between very closely related organisms, these methods likely have a high rate of false-positive and negative predictions (8,9). Moreover, GC composition within the same genome may fluctuate considerably for different genomic regions (10,11) and (even) for some vertically inherited genes (e.g. ribosomal proteins) (12). In turn, implicit phylogenetic methods are limited by their reliance on similarity scores and underlying phylogeny. This poses another problem since statistically significant sequence similarity is not necessarily a result of vertical evolution (13,14). Because genes acquired from foreign sources typically do not show congruence to species trees, one way to detect HGT would be to reconcile gene trees against reference species trees. This principle is based on an explicit evolutionary model and is generally considered to be a reliable alternative to detect HGT events (15). However, its practical use has remained limited because reconciling trees is computationally intensive (13) and because tree incongruence can also arise from processes other than HGT (16) (see Discussion).

Here, we introduce HGTree (http://hgtree.snu.ac.kr) that provides putative genome-wide HGT information for 2472 completely sequenced prokaryotic genomes. HGTree defines HGT by comparing the gene tree for each orthologous gene set to the reference species tree. Conflict between gene and species trees is taken as indication of non-vertical evolution. Specifically, different hypotheses regarding the evolution of gene sets are evaluated and only those corresponding to HGT are kept and stored in the database. Results are displayed graphically for quick understanding. The friendly user-interface allows quick retrieval of already processed results for HGT analysis. Currently, three major services are provided: (i) HGT browser to display the molecular functions, gene family and phylogenetic relationships of HGT-acquired genes for all the genomes in the database, (ii) HGT analysis between and within (user selected) genomes and (iii) HGT analysis of user submitted gene and genome sequences. For each service, donor and recipient genomes are also graphically labeled for quick understanding. The database is freely available, does not require registration or login credentials and can be easily scaled and updated to keep pace with the continuous rise in genomic information. Importantly, HGTree represents the most complete existing resource for HGT-related information built on an explicit evolutionary model of tree reconciliation.

## MATERIALS AND METHODS

### Data retrieval

**Figure 1.**
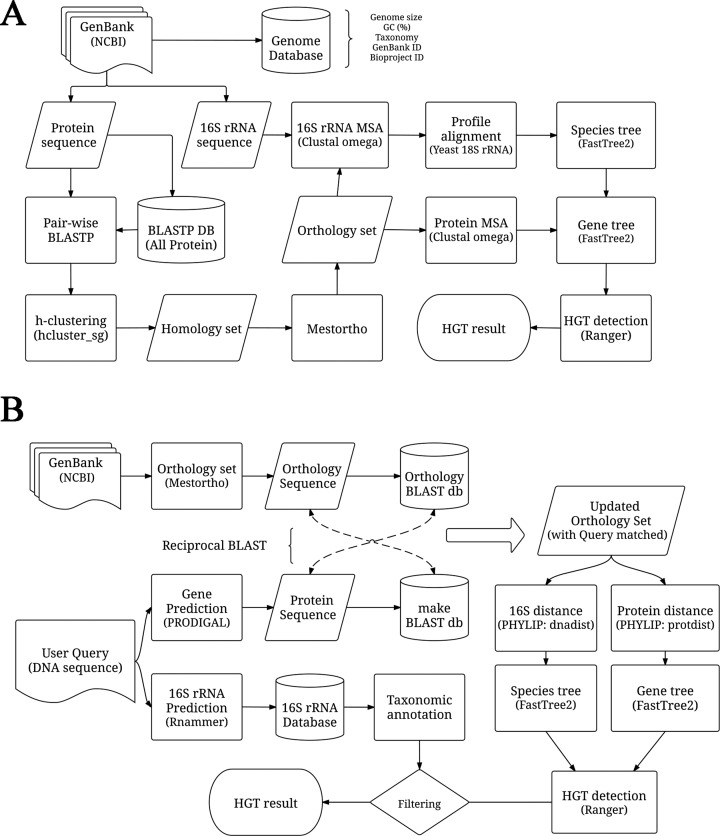
Workflow of the HGTree analysis pipeline.**(A)** HGT-detection in prokaryotic genomes. **(B)** Pipeline to process user gene and genome data. See Materials and Methods and main text for detailed description and filtering criteria.

Genome data were retrieved from NCBI using ‘prokaryote’ and ‘complete’ search options (http://www.ncbi.nih.gov/Genomes/; 17 March, 2015) (17). After removing redundant genomes, a total of 2472 completely sequenced prokaryotic genomes (156 Archaea and 2316 Bacteria; Supplementary Table S1) were selected for downstream processing. From each GenBank file (18), information regarding taxonomy, GC content (%), GenBank and Bioproject IDs, genome size, nucleotide and amino acid sequences, gene symbol and gene function were either extracted or calculated (Figure 1A). Out of the total 2472 genomes, 30 belonged to human microbiota (19) (Table 1).

**Table 1.** Summary statistics

Type	Number of records
Total non-redundant microbial genomes	2472^a^
Genomes part of human microbiota	30
Total protein sequences	7 748 306
Number of orthologous gene sets	154 805
Detected putative HGT events	660 840

^a^156 Archaea and 2316 Bacteria.

### Functional annotation

A total of 7 748 306 genes in 2472 genomes were scanned against Clusters of Orthologous Genes (COG) database (20) using HMMER (ver. 3.0) (*E*-value < 10^−3^) (8). Protein family level assignments were calculated using the local installation of PfamScan (ver. 1.3) following default parameters (21). RNammer (ver.1.2) (22) was used to detect 16S rRNA sequences in each genome. The set of orthologous genes in each species was mapped to corresponding 16S rRNA sequence and this information was used to determine the conflict between gene and species trees during downstream processing.

### Orthology assignment

Ensembl homology prediction pipeline (23) was implemented to define homologous gene sets (Figure 1A). First, pairwise BLASTP (24) search was conducted on each protein from every genome against the total set of proteins (both self and non-self species). For this step, BLAST hits were required to have alignment coverage of at least 80% for both query and subject as well as stringent *E*-value cutoff of 10^−6^. Second, a sparse graph was built that described relationships between genes based on BLAST results. Third, homologous clusters were generated using hcluster_sg (25) program (ver. 0.5.1) that clusters sequences in an hierarchical manner by considering the mean distance between sequences. Fourth, based on homology information, orthologous gene sets were predicted using a modified version of Mestortho orthology detection algorithm (ver. 2.0) (26) optimized to work with large data sets. To improve precision, we removed following orthologous groups from the analysis: (i) gene sets containing >50% of the total genomes since their inclusion contributed towards greater computational load, (ii) gene sets with less than four operational taxonomic units (OTUs) since it is the minimum requirement to build an un-rooted phylogenetic tree and (iii) gene sets consisting of only one species due to the presence of several type-strains that could not be distinguished by 16S rRNA analysis.

### Tree reconstruction

Multiple sequence alignment (MSA) of orthologous gene sets was generated using CLUSTAL Omega (ver.1.2.1) (27) under default settings (Figure 1A). 16S rRNA sequences extracted from each genome were also aligned in a similar way and then combined into a profile alignment along with 18S rRNA sequence from *Saccharomyces cerevisiae*. The eukaryotic rRNA sequence was treated as outgroup to root the species tree and was removed once Newick trees were produced. Pair-wise distance matrices were calculated for MSAs of both orthologous gene sets and corresponding 16S rRNA sets. Orthologous gene sets where all pair-wise distances between proteins were close to zero (< 0.0001) were removed, as they do not provide enough information for reliable estimation of phylogenetic relationships. FastTree (ver. 2.0) was used to reconstruct phylogenetic trees for each orthologous gene set and the corresponding species tree (28). FastTree calculates approximate maximum-likelihood (ML) trees by first building a starting neighbor-joining (NJ) tree and then refining it by a combination of minimum evolution and maximum-likelihood approaches (28). It is much faster than the standard ML-based programs such as PhyML 3 (29) and RAxML (30) and is optimized to work with large data sets while ensuring high accuracy (28). Species tree was re-rooted by the yeast sequence *a posteriori* using the Newick Utility (ver. 1.6) (31). The reliability of splits in phylogenetic trees was evaluated by ‘local support values’ based on Shimodaira-Hasegawa (SH) test (32) similar to ‘SH-like local support’ values in PhyML 3. RANGER-DTL-U (ver.1.0) (33) was used to detect putative HGT events by reconciling gene trees against rooted 16S rRNA reference species tree and to distinguish HGT events from gene duplication and loss events (Figure 1A). All HGT events except those between same species were stored along with species and gene information.

### Processing of user queries

User submitted sequences are processed in the following manner: (i) Prodigal (ver. 2.6) is used to detect protein-coding genes; (ii) orthologous groups are assigned to predicted genes by searching against already constructed orthologous gene sets using reciprocal-BLAST search (Figure 1B). Several measures are taken to ensure reliable assignment of orthologous groups to user-provided sequences including alignment coverage of at least 80% between query and subject, stringent *E*-value threshold of < 10^−6^ and enabling soft-masking (18); (iii) orthologous groups that contain user queries form updated orthology sets; (iv) in parallel, Rnammer is used to detect 16S rRNA sequences; (v) user-provided 16S rRNA sequences are searched against the 16S rRNA database constructed previously from 2472 prokaryotic genomes to predict the taxonomic structure of user-provided data; (vi) MSA, distance matrices, filtering and phylogenetic trees are calculated as described above using the updated orthology set and 16S rRNA information. However, users may opt to select a different outgroup taxon for rooting the species tree depending upon their preferences. This can be accomplished by providing the new 16S (18S) rRNA sequence and selecting the appropriate option on the online menu. By default, user data is processed via FastTree. However, ML based processing of user queries can be up to three times slower than NJ processing (Table 2). Therefore, we provide an alternative option to quickly process user queries using NJ tree reconstruction from pre-computed distance matrices and (vii) HGT events corresponding to user sequences are extracted and returned to user by *E*-mail.

**Table 2.** Processing time required for genomes of varying sizes.

Genome	GS^a^ (Mb)	NP^b^	NJ^c^ (min)	ML^d^ (min)
*Candidatus Nasuia*	0.11	137	1.69	1.67
*Mycoplasma gallisepticum*	1.01	753	4.43	4.71
*Chlamydia psittaci*	1.18	972	12.74	23.23
*Bartonella quintana*	1.58	1206	12.30	35.20
*Bifidobacterium animalis*	1.93	1530	16.92	30.92
*Zymomonas mobilis*	2.06	1750	17.06	39.93
*Corynebacterium urealyticum*	2.37	1953	19.16	44.75
*Staphylococcus warneri*	2.49	2298	26.58	83.07
*Methanoregula formicica*	2.82	2775	20.85	55.80
*Psychromonas*	3.05	2559	41.78	95.82
*Legionella pneumophila*	3.4	2943	35.14	68.03
*Gluconobacter oxydans*	3.6	3197	25.01	43.03
*Janthinobacterium*	4.11	3770	36.64	77.86
*Alteromonas macleodii*	4.44	3800	43.28	109.25
*Stenotrophomonas maltophilia*	4.85	4354	46.68	79.82
*Azotobacter vinelandii*	5.37	4660	54.64	124.01
*Microcoleus*	7.97	6003	43.56	89.64
*Niastella koreensis*	9.03	7136	50.75	89.85
*Myxococcus stipitatus*	10.35	7949	55.71	88.94
*Sorangium cellulosum*	13.03	9445	56.69	87.51

^a^Genome Size.

^b^Number of protein coding sequences.

^c^Processing time using NJ.

^d^Processing time using ML.

### Statistical test to detect HGT enriched phyla

Fisher's exact test was performed to test the significance of the null hypothesis stating that HGT events of a particular phylum were not greater compared to other phyla. For this purpose, 2 × 2 contingency tables for each phylum were analyzed. Specifically, the counts of HGT-related and total genes for each phylum were compared with the counts of HGT-related and total genes in all other phyla. The odds ratio greater than one favored the alternate hypothesis stating that HGT events of a particular phylum were significantly greater than the HGT events of all other phyla.

### Database server and user interface

The database server was developed using MariaDB (ver.10.0.13) (http://mariadb.org/) management system. The database consists of four tables with more than 13 million records. The web-based user interface was written in HTML5, PHP, CSS and JavaScript. User interface widgets were implemented using jQWidgets (ver.3.8.1) (http://www.jqwidgets.com) and jQuery (ver.1.11) (http://jquery.com). Circular phylogenetic trees were generated by jsPhyloSVG-1.55 (34) and two way HGT relationships (donors and recipients) were dynamically generated using the SVG JavaScript library, D3 (35).

## RESULTS

### Organization of HGTree

The interface of HGTree consists of six main menus: *Home*, *Background*, *Search*, *Downloads*, *Tutorial* and *Contact us*. *Home* is the welcome window providing easy navigation to other menus and contains basic information about the database. *Background* gives the rationale behind the development of HGTree and schematically describes the HGT detection process. *Search* consists of five sub-menus: (i) HGT Browser, (ii) HGT Analysis within Selected Genomes, (iii) Between-group HGT Analysis, (iv) HGT Analysis of User Query and (v) Gene or Keyword Search. Each of the sub-menus is described below. In addition, users can download FASTA formatted protein and 16S rRNA sequences, general description files for each genome, and pre-computed alignments and phylogenetic trees corresponding to all genes and species sets from the *Downloads* menu. To facilitate easy navigation and understanding, step-by-step tutorials are also available from the *Tutorial* menu.

**Figure 2.**
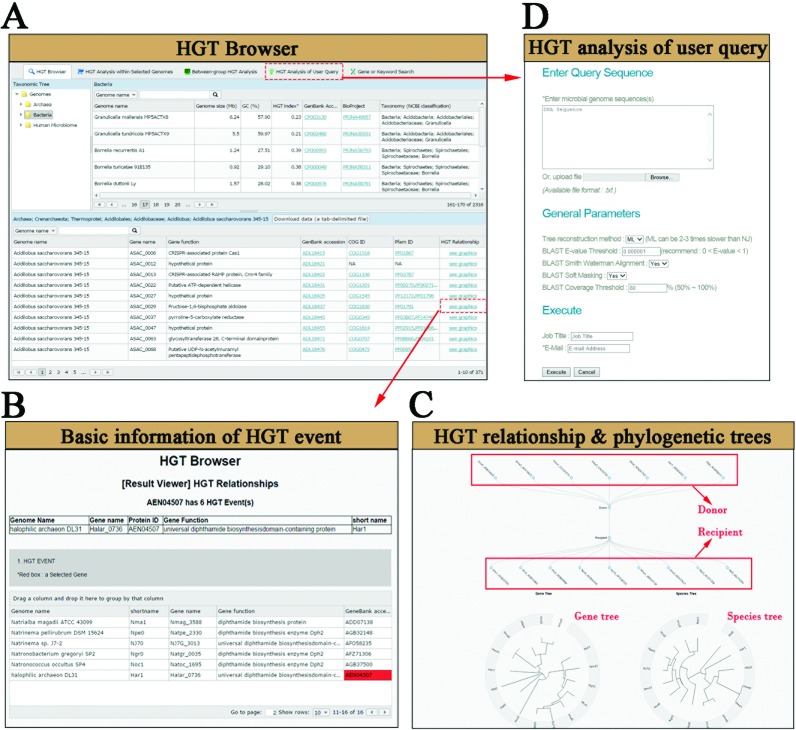
Screenshots of **HGT Browser** functionality in HGTree. **(A)** Users can either search for their genome of interest or navigate through the ‘Taxonomic Tree’. Upon selection of genome(s), list of HGT-related genes are displayed at the bottom. **(B)** Tables display basic information about all genes that have participated in HGT events. **(C)** Plots display donors and recipient genomes in each HGT event, as well as both gene and species trees. **(D)** Users can query their gene or genome sequences against our servers to identify HGT-related genes in their data.

**HGT Browser** gives complete information related to all genomes and HGT events stored in the database. The current version of HGTree contains a total of 660 894 HGT events detected in 2472 microbial genomes (Supplementary Table S2). A search box allows users to search for their genome of interest. Alternatively, users may navigate from the classification window provided on the left under ‘Taxonomic Tree’ (Figure 2A). For each selected organism, genome size, GC content (%), GenBank and BioProject IDs and complete taxonomic information are also displayed (see also Supplementary Table S2). In addition, we provide an HGT-index that is a quantitative indicator of HGT influence in each genome. The index simply represents the total number of HGT-related genes (both donor and recipient) divided by the total number of genes in a genome. The table directly below lists all HGT events detected in the selected genome(s) (Figure 2A). For each event, several links provide access to Pfam and COG classifications along with basic description of gene function. HGT events and phylogenetic relationships can be visualized graphically to explicitly highlight the conflict between gene and species trees. For example, clicking ‘see graphics’ under ‘HGT Relationship’ column will return graphical representation of HGT relationships with other microbial genomes (Figure 2B). Plots show donor and recipient genomes involved in each HGT event as well as gene and species trees (Figure 2C). Trees can be displayed either in circular or rectangular representation. The latter also displays the local support values to provide a quick estimate for the reliability of phylogenetic splits. By default, HGTree displays ML gene and species trees as inferred by FastTree (28). However, we also reconstructed NJ trees separately for each gene and corresponding species set. All HGT-related information can be downloaded from the *Downloads* menu.

While HGT Browser can be used to find genome-wide HGT events of a genome against all other genomes, **HGT Analysis within Selected Genomes** utility can display HGT events that have occurred only within selected genomes. For this analysis, users are prompted to select at least two different species regardless of their phyla classification. This exercise can be useful to quantify gene flow between species that maybe engaged in symbiosis-like relationships. In turn, **Between-group HGT Analysis** tool enables users to customize two groups of organisms. Users may add organisms from different phyla in each group. The analysis option then displays HGT events that have occurred between the user-defined groups. For example, obtaining group-wise HGT information can be useful to understand interactions between different microbial phyla in environmental samples. HGTree also offers users to detect HGT events in their own gene or genome sequences (Figure 2D). For this purpose, users may upload FASTA formatted DNA sequences that are scanned against the pre-compiled data sets (as described above) for fast NJ reconstruction. Alternatively, users may opt to process results using FastTree approximate ML trees (28), as we have done throughout the database. However, ML-based processing is about 2–3 times slower relative to NJ reconstruction (Table 2) because the distance matrices required for NJ reconstruction are already pre-computed and simply need to be updated with user data. In addition, analysis time depends upon genome length, total number of proteins and number of genes matched to orthologous gene sets. On average analyzing 1MB genome and 1000 proteins roughly takes 9 and 17 minutes under NJ and ML processing, respectively, on background computing server equipped with 8 CPU cores (16 processors @ 2.60 GHz and 128 GB RAM) (Table 2). For example, it took less than 2 minutes to process the smallest genome in our data set (Candidatus *Nasuia*; 110KB) using both NJ and ML. In turn, it took 57 and 88 minutes to process the largest genome (*Sorangium cellulosum*; 13.03 MB) under NJ and ML environments respectively (Table 2). Therefore, users can opt for either option depending upon their convenience. Results are processed on our servers and returned to users via *E*-mail. Finally, users may opt to study the evolution of a particular gene family. This can be accomplished by typing either the gene name or its function (e.g. CRISPR) using the **Gene or Keyword Search** utility provided by the *Search* menu.

### Initial insights into microbial evolution

**Figure 3.**
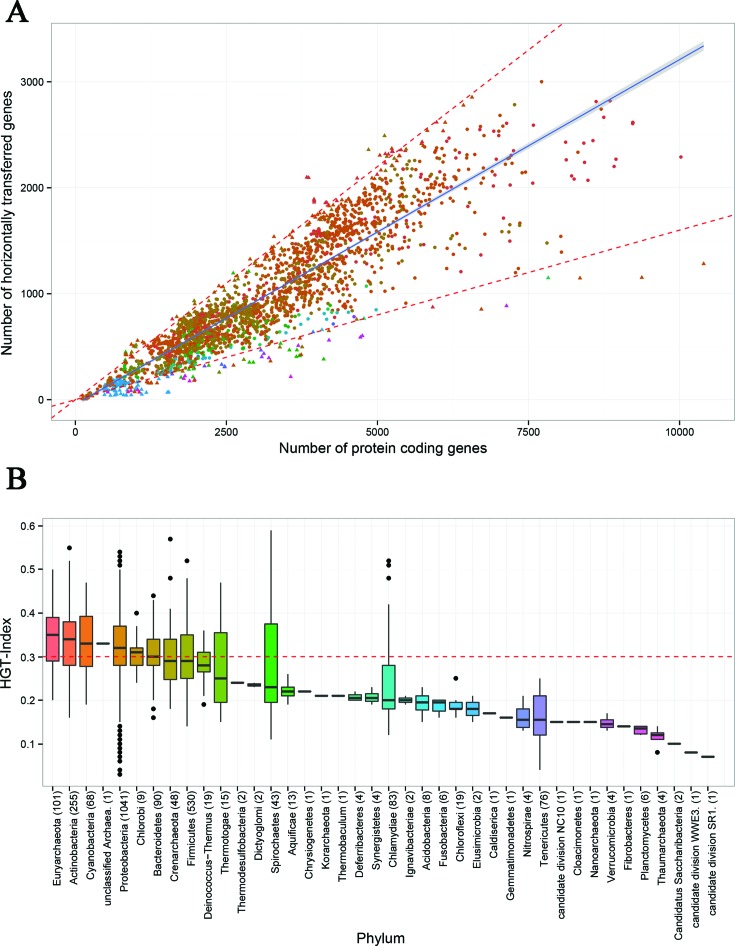
Microbial genomes as viewed by HGTree. **(A)** Each triangle in the scatter-plot represents one microbial genome. The fitted regression line (blue) (*y* = −44.31 + 0.33X; *R*^2^ = 0.81) describes a linear relationship between the number of HGT-related genes and the total number of genes in each genome. The gray area around the regression line indicates standard error. The red-dotted line excludes organisms that fall in the upper and lower 5% percentiles of HGT-index. **(B)** Boxplots show the distribution of HGT-index values for organisms in each major microbial phylum in our data set. The horizontal red line represents the global median HGT-index value (0.3). Phyla are sorted in descending order based on their median HGT-index. Numbers in parenthesis indicate total number of genomes sampled for each phylum/group.

HGTree already provides preliminary insights into microbial evolution. The data suggest abundance of genetic exchange among microbial species (Figure 3). The HGT-index ranges from 0.03 (Candidatus *Hodgkinia* and *Mycoplasma haemofelis*) to 0.59 (*Borrelia garinii*) (Supplementary Table S2). While most microbial genomes showed linear relationship between the total number of genes and total number of horizontally transferred genes, some outlier genomes with significantly (*P* < 0.05) lower or higher HGT-index were also detected. Specifically, we focused on the 5% upper (HGT-index < 0.16) and lower (HGT-index > 0.42) percentiles of HGT-index as shown in the red-dotted line in Figure 3A (see also Supplementary Table S2). Among 247 outlier genomes, *Chlamydia*, *Rickettsia* and *Mycobacterium* genera belonged to the upper percentile, while *Mycoplasma* to the lower. Interestingly, these organisms are notable parasites of other species suggesting that symbiosis and parasitism lead to significant increase/decrease in horizontal exchange (36). Figure 3B gives a breakdown of HGT influence in each major microbial phylum. We observed that five phyla (Euryachaeota, Actinobacteria, Cyanobacteria, Proteobacteria and Chlorobi) and one unclassified archaeon had relatively higher HGT-index than the global median value of 0.3 (red dashed line in Figure 3B). Fisher's exact test confirmed that Euryachaeota (101 members), Actinobacteria (255), Cyanobacteria (68) and Proteobacteria (1041) were significantly (*P*-value < 0.05) enriched by HGT. In turn, Chlorobi (9 members) and the unclassified archaeon (1) were deemed ‘statistically insignificant’ despite HGT-index above the global median. Because the Fisher's test calculates HGT enrichment by relating the number of HGT-genes and total number of genes for one phylum against all other phyla (the background), poorly sampled lineages likely contributed little information relative to the background. Similarly, the HGT-index must also be interpreted with caution as it depends upon the total number of ‘known’ genes in each phylum that could be matched to orthologous gene sets. Because poorly sampled lineages likely contain many ‘rare’ genes with either no or few orthologs in sequence databases, their HGT-index was lower relative to well-studied lineages in our data set (albeit with some exceptions; Figure 3B). Importantly, the two most well sampled bacterial phyla (Proteobacteria and Firmicutes) had median HGT-index of 0.32 and 0.29, respectively. These numbers suggest that while HGT was likely underestimated for poorly sampled lineages, its median upper-bound still lies somewhere around 0.3 and 0.4. Previously, Dagan et al. (2008) estimated that on average 81 ± 15% genes in the genomes of 181 prokaryotic species had participated in horizontal exchange (37). In turn, our results reveal that HGT-index in most microbial phyla, especially those that are well studied and sampled, did not reach extremely high levels. In fact, HGT-index suggests that about 10–35% of genes in most microbial phyla are subject to horizontal exchange (Figure 3B). The differences between two studies are likely due to two main reasons: (i) increased sampling of microbial genomes in this study (2472 versus 181) and (ii) an explicit evolutionary model backs the detection of HGT-related genes. In turn, Dagan et al. (2008) did not consider phylogenetic discordance. This should be kept in mind when comparing the two studies. The results however confirm current understanding that HGT plays significant roles in the evolution of microbial organisms and must be closely monitored for both medical and economical purposes.

## DISCUSSION

HGTree is based on an explicit evolutionary model i.e. conflict between gene and species trees is taken as indication of non-vertical evolution. In general, evaluating incongruence between gene and species trees holds promise to reliably detect HGT events (e.g. see (15)). However, its practical use has remained limited because, (i) the choice of tree reconstruction method (e.g. NJ, ML, parsimony) can influence HGT detection, (ii) accurate detection of orthology remains a challenge, (iii) conflicts between gene and species trees may also arise from processes other than HGT such as reductive evolution (16) and (iv) tree reconstruction followed by reconciliation are computationally intensive tasks. These considerations make it technically and conceptual challenging to globally infer HGT events (i.e. by reconciling trees for all gene families in hundreds of organisms). Below, we describe measures taken to ensure that HGTree was minimally affected from these challenges.

To ensure high speed and optimal accuracy in tree reconstruction, we implemented FastTree program to infer approximate ML phylogenies for each orthologous gene set and its corresponding species tree (28). FastTree is more than 100 times faster than the standard ML programs (PhyML 3.0 and RAxML 7) and is significantly more accurate than distance and parsimony based methods of tree reconstruction (28). It even outperforms the default implementation of PhyML 3 but is less accurate than PhyML and RaxML ran with subtree-pruning-regrafting (SPR) options. However, this is more than offset by the speedier execution of FastTree in handling large alignments containing hundreds of taxa. Moreover, disagreements between FastTree and SPR-based ML programs tend to be poorly supported (28). FastTree also provides local support values based on SH test (32) to quickly evaluate the reliability of obtained trees. These values correlate well with the SH-like support values provided by PhyML 3 (28) and can be used to quickly determine the reliability of inferred phylogenetic splits. In turn, running traditional bootstrap would considerably increase the processing time plus adding the time for tree reconciliation. These features identified FastTree as the optimal choice to rapidly and accurately reconstruct hundreds of phylogenetic trees in our data set.

To accurately define orthologs, we incorporated Mestortho, which is an orthology detection algorithm based on minimum evolution (26). To improve precision in orthology estimation, we filtered out gene sets exhibiting either high or low complexity (see Materials and Methods). To evaluate conflicting hypotheses regarding the evolution of gene sets, RANGER-DTL-U was used to reconcile unrooted gene trees against rooted species trees and to postulate gene duplication, transfer and loss events (commonly known as DTL reconciliation) (see (33) and references therein). The algorithm works by embedding each possible rooted version of gene tree inside the species tree and selecting the most parsimonious reconciliation amongst all rootings (i.e. explain the transformation of gene tree into species tree with minimum overall cost). Thus, RANGER-DTL is built on parsimony principle similar to most existing algorithms of tree reconciliation (e.g. (38–41)), except (42) and (43) that utilize probabilistic framework. However, RANGER-DTL significantly outperforms others when dealing with huge data sets containing trees of hundreds of taxa (33). In a comparative exercise, it was sometimes 100 000 times faster than Mowgli (41) and AnGST (40), two other widely used advanced algorithms for DTL reconciliation. An alternative version of the program (RANGER-DTL-D) requires dated species trees (i.e. chronogram) for reconciliation. While, the alternative is biologically well founded and considers HGT to only occur between co-existing species, accurate estimation of dates for each and every phylogenetic tree currently remains challenging, especially for large trees (44). Moreover, it is relatively much slower for large data sets (33). In turn, most other available reconciliation algorithms consider duplication and loss but not transfers (e.g. (38,45,46)) and hence are not suitable for large-scale analyses of prokaryotic gene phylogenies. Therefore, RANGER-DTL-U is implemented in the current version of HGTree due to its speed, accuracy and compatibility in handling large data sets.

HGTree is a non-commercial public database developed to support various fields of research. It has a user-friendly interface allowing easy access to large amount of HGT information. To our knowledge, it is the most comprehensive available resource of HGT-related information generated by large-scale phylogenetic analyses. However, not all transfers can be detected by tree reconciliation. For example, transfers that occur between sister taxa do not yield topological incongruence. Similarly, an HGT-acquired gene in the common ancestor of two (or more) species may later be lost in only one (or more) of the descendants. Here, favoring either transfer or loss can be conceptually challenging because the gene has experienced both events. The decision to record the event either as HGT or to ignore (i.e. treat as loss) is based on the most parsimonious embedding of gene tree inside the species tree. In our opinion, recording such events as transfers is more appropriate because HGT followed by loss nullifies the first gain and restores the original state. Technically, such events could still be recorded as losses if they yield the most parsimonious reconciliation and hence will be excluded from the HGTree repository. In other words, some true positives have likely been missed and false-positives included thanks to the biological complexity of the HGT-detection problem. We expect to quantify these rates in a future version. Because tree reconciliation method is susceptible to topologies of both species and gene trees, short branch lengths of species tree may also sometimes lead to incorrect estimation of HGT. However, no method for HGT detection can be 100% accurate. Therefore, genes output by HGTree should be taken as putative HGT-genes to aid further downstream analysis. Moreover, results are dependent upon the choice and accuracy of existing programs for tree reconstruction and reconciliation and will no doubt improve in precision with the availability of better alternatives in future.

### Future work

The precision and use of HGTree can be improved with additional upgrades. In the present version, we removed orthologous gene sets containing >50% of total microbial genomes. While some widely distributed gene families may also be subject to HGT (e.g. aminoacyl-tRNA synthetase), their accurate detection via phylogenetic inferences can be more challenging. In turn, these transfers can perhaps be better detected via comparative genomics approaches, as shown in (47). Moreover, large gene families contributed to maximum computational load in HGT detection. Therefore, we plan to equip HGTree with surrogate measures of HGT detection to take care of these issues. Similarly, when there is consistent HGT signal between donor and recipient lineages, concatenating such genes may give better resolution. However, concatenated genes can be subject to other artefacts. For example, genes are composed of protein domains that can be gained, lost or rearranged in genes (48). Their inclusion in sequence alignments can increase the number of gaps and thus artificially influence phylogenetic inferences. We expect to reconcile concatenated gene phylogenies against individual gene phylogenies in the subsequent releases to better address this issue. Another issue related to reconciling trees is the existence of multiple optimal reconciliations that may be equally good. The similarities and differences between multiple optimal solutions were recently explored on a biological data set of ≈4700 gene trees reconciled against species tree (49). The authors confirmed that despite the existence of multiple optimal solutions, event assignments to gene nodes and mappings were fairly conserved across all optimal solutions (e.g. 93.1% and 73.15% chances for events and mappings respectively) (49). Unfortunately, exploring optimal search space and listing percentages of conserved events is not part of the current release of RANGER-DTL but an update is expected soon. Therefore, we expect to provide numeric confidence to each event assignment in the future releases of HGTree provided that search space can be explored in reasonable amount of time. The precision will also improve with the availability of high quality genome assemblies and sequencing of novel organisms. The future versions will also focus on detection of HGT-derived gene clusters in microbial genomes since transfer of gene clusters is a frequent event in microbial evolution (50). Viral genomes will also be added in the subsequent releases, as viruses often exchange/transfer genes between microbial species (51). Finally, HGT contribution of microbial species that are part of normal human microbiota will also yield useful insights into the complex ways organisms interact with each other (19). We expect to update HGTree at least twice a year to keep pace with the rising genomic information.

## References

[1] Woese C.R. (1987). Bacterial evolution. Microbiol. Rev..

[2] Magiorkinis G., Belshaw R., Katzourakis A. (2013). 'There and back again': revisiting the pathophysiological roles of human endogenous retroviruses in the post-genomic era. Philos Trans R Soc Lond B Biol Sci..

[3] Doolittle W.F. (1999). Phylogenetic classification and the universal tree. Science.

[4] Salyers A.A., Gupta A., Wang Y. (2004). Human intestinal bacteria as reservoirs for antibiotic resistance genes. Trends Microbiol..

[5] Koonin E.V., Makarova K.S., Aravind L. (2001). Horizontal gene transfer in prokaryotes: quantification and classification 1. Annu. Rev. Microbiol..

[6] Garcia-Vallvé S., Guzmán E., Montero M., Romeu A. (2003). HGT-DB: a database of putative horizontally transferred genes in prokaryotic complete genomes. Nucleic Acids Res..

[7] Podell S., Gaasterland T., Allen E.E. (2008). A database of phylogenetically atypical genes in archaeal and bacterial genomes, identified using the DarkHorse algorithm. BMC Bioinformatics.

[8] Lawrence J.G., Ochman H. (2002). Reconciling the many faces of lateral gene transfer. Trends in microbiology.

[9] Guindon S., Perriere G. (2001). Intragenomic base content variation is a potential source of biases when searching for horizontally transferred genes. Mol. Biol. Evol..

[10] Deschavanne P., Filipski J. (1995). Correlation of GC content with replication timing and repair mechanisms in weakly expressed E. coli genes. Nucleic Acids Res..

[11] Wuitschick J.D., KARRER K.M. (1999). Analysis of genomic G+ C content, codon usage, initiator codon context and translation termination sites in Tetrahymena thermophila. J. Eukaryot. Microbiol..

[12] Muto A., Osawa S. (1987). The guanine and cytosine content of genomic DNA and bacterial evolution. Proc. Natl. Acad. Sci. U.S.A..

[13] Ravenhall M., Škunca N., Lassalle F., Dessimoz C. (2015). Inferring horizontal gene transfer. PLoS Comput. Biol..

[14] Koski L.B., Golding G.B. (2001). The closest BLAST hit is often not the nearest neighbor. J. Mol. Evol..

[15] Ragan M.A. (2001). On surrogate methods for detecting lateral gene transfer. FEMS Microbiol. Lett..

[16] Than C., Ruths D., Innan H., Nakhleh L. (2007). Confounding factors in HGT detection: statistical error, coalescent effects, and multiple solutions. J. Comput. Biol..

[17] NCBI Resource Coordinators (2015). Database resources of the National Center for Biotechnology Information. Nucleic Acids Res..

[18] Benson D.A., Clark K., Karsch-Mizrachi I., Lipman D.J., Ostell J., Sayers E.W. (2015). GenBank. Nucleic Acids Res..

[19] Human Microbiome Project Consortium (2012). Structure, function and diversity of the healthy human microbiome. Nature.

[20] Galperin M.Y., Makarova K.S., Wolf Y.I., Koonin E.V. (2014). Expanded microbial genome coverage and improved protein family annotation in the COG database. Nucleic Acids Res..

[21] Punta M., Coggill P.C., Eberhardt R.Y., Mistry J., Tate J., Boursnell C., Pang N., Forslund K., Ceric G., Clements J. (2012). The Pfam protein families database. Nucleic Acids Res..

[22] Lagesen K., Hallin P., Rødland E.A., Stærfeldt H.-H., Rognes T., Ussery D.W. (2007). RNAmmer: consistent and rapid annotation of ribosomal RNA genes. Nucleic Acids Res..

[23] Vilella A.J., Severin J., Ureta-Vidal A., Heng L., Durbin R., Birney E. (2009). EnsemblCompara GeneTrees: Complete, duplication-aware phylogenetic trees in vertebrates. Genome Res..

[24] Camacho C., Coulouris G., Avagyan V., Ma N., Papadopoulos J., Bealer K., Madden T.L. (2009). BLAST+: architecture and applications. BMC Bioinformatics.

[25] Li H., Coghlan A., Ruan J., Coin L.J., Heriche J.-K., Osmotherly L., Li R., Liu T., Zhang Z., Bolund L. (2006). TreeFam: a curated database of phylogenetic trees of animal gene families. Nucleic Acids Res..

[26] Kim K.M., Sung S., Caetano-Anollés G., Han J.Y., Kim H. (2008). An approach of orthology detection from homologous sequences under minimum evolution. Nucleic Acids Res..

[27] Sievers F., Wilm A., Dineen D., Gibson T.J., Karplus K., Li W., Lopez R., McWilliam H., Remmert M., Söding J. (2011). Fast, scalable generation of high-quality protein multiple sequence alignments using Clustal Omega. Mol. Syst. Biol..

[28] Price M.N., Dehal P.S., Arkin A.P. (2010). FastTree 2–approximately maximum-likelihood trees for large alignments. PloS One.

[29] Guindon S., Delsuc F., Dufayard J.-F., Gascuel O. (2009). Estimating maximum likelihood phylogenies with PhyML. Methods Mol. Biol..

[30] Stamatakis A. (2006). RAxML-VI-HPC: maximum likelihood-based phylogenetic analyses with thousands of taxa and mixed models. Bioinformatics.

[31] Junier T., Zdobnov E.M. (2010). The Newick utilities: high-throughput phylogenetic tree processing in the UNIX shell. Bioinformatics.

[32] Shimodaira H., Hasegawa M. (1999). Multiple comparisons of log-likelihoods with applications to phylogenetic inference. Mol. Biol. Evol..

[33] Bansal M.S., Alm E.J., Kellis M. (2012). Efficient algorithms for the reconciliation problem with gene duplication, horizontal transfer and loss. Bioinformatics.

[34] Smits S.A., Ouverney C.C. (2010). jsPhyloSVG: a javascript library for visualizing interactive and vector-based phylogenetic trees on the web. PloS One.

[35] Bostock M., Ogievetsky V., Heer J. (2011). D^3^ data-driven documents. IEEE Trans. Vis. Comput. Graph..

[36] Nasir A., Naeem A., Khan M.J., Nicora H.D.L., Caetano-Anollés G. (2011). Annotation of protein domains reveals remarkable conservation in the functional make up of proteomes across superkingdoms. Genes.

[37] Dagan T., Artzy-Randrup Y., Martin W. (2008). Modular networks and cumulative impact of lateral transfer in prokaryote genome evolution. Proc. Natl. Acad. Sci. U.S.A..

[38] Charleston M. (1998). Jungles: a new solution to the host/parasite phylogeny reconciliation problem. Math. Biosci..

[39] Conow C., Fielder D., Ovadia Y., Libeskind-Hadas R. (2010). Jane: a new tool for the cophylogeny reconstruction problem. Algorithms Mol. Biol..

[40] David L.A., Alm E.J. (2011). Rapid evolutionary innovation during an Archaean genetic expansion. Nature.

[41] Doyon J.-P., Hamel S., Chauve C. (2012). An efficient method for exploring the space of gene tree/species tree reconciliations in a probabilistic framework. IEEE/ACM Trans. Comput. Biol. Bioinform..

[42] Tofigh A. (2009). Using trees to capture reticulate evolution, lateral gene transfers and cancer progression.

[43] Csűrös M., Miklós I. (2009). Streamlining and large ancestral genomes in Archaea inferred with a phylogenetic birth-and-death model. Mol. Biol. Evol..

[44] Rutschmann F. (2006). Molecular dating of phylogenetic trees: a brief review of current methods that estimate divergence times. Divers. Distrib..

[45] Eulenstein O., Vingron M. (1998). On the equivalence of two tree mapping measures. Discrete Appl. Math..

[46] Page R.D. (1994). Maps between trees and cladistic analysis of historical associations among genes, organisms, and areas. Syst. Biol..

[47] Nasir A., Caetano-Anollés G. (2013). Comparative analysis of proteomes and functionomes provides insights into origins of cellular diversification. Archaea..

[48] Nasir A., Kim K.M., Caetano-Anollés G. (2014). Global patterns of protein domain gain and loss in superkingdoms. PLoS Comput. Biol..

[49] Bansal M.S., Alm E.J., Kellis M. (2013). Reconciliation revisited: handling multiple optima when reconciling with duplication, transfer, and loss. J. Comput. Biol..

[50] Ochman H., Lawrence J.G., Groisman E.A. (2000). Lateral gene transfer and the nature of bacterial innovation. Nature.

[51] Weinbauer M.G., Rassoulzadegan F. (2004). Are viruses driving microbial diversification and diversity?. Environ. Microbiol..

